# Rapid increase of resistance to erythromycin and clindamycin in Streptococcus pyogenes in Italy, 1993-1995. The Italian Surveillance Group for Antimicrobial Resistance.

**DOI:** 10.3201/eid0204.960410

**Published:** 1996

**Authors:** G Cornaglia, M Ligozzi, A Mazzariol, M Valentini, G Orefici, R Fontana

**Affiliations:** Institute of Microbiology, University of Verona, Italy.

## Abstract

A survey of antibiotic resistance in Streptococcus pyogenes in Italy showed a sharp increase in erythromycin resistance. In 1993, the incidence of erythromycin-resistant strains was on average 5.1%, with marked variations by geographic area. Two years later, the incidence of these strains had registered a 1.5- to roughly 20-fold increase, with a mean value of 25.9%, exceeding 40% in three centers out of 13 and 30% in another four. For all the strains studied, normal levels of susceptibility to penicillin were reported.


### Rapid Increase of Resistance to Erythromycin and Clindamycin in *Streptococcus pyogenes* in Italy, 1993-1995

A survey of antibiotic resistance in *Streptococcus pyogenes* in Italy showed a sharp increase in erythromycin resistance. In 1993, the incidence of erythromycin-resistant strains was on average 5.1%, with marked variations by geographic area. Two years later, the incidence of these strains had registered a 1.5- to roughly 20-fold increase, with a mean value of 25.9%, exceeding 40% in three centers out of 13 and 30% in another four. For all the strains studied, normal levels of susceptibility to penicillin were reported.

Over the past few years, the increased frequency of infections caused by *Streptococcus pyogenes* (group A streptococcus [GAS]) and their sequelae has been reported in several parts of the world (1,2). Even though these reports may reflect an enhanced awareness of and interest in these possibly life-threatening infections on the part of the medical community (3), in at least some areas, an increase in severe infections over time has been documented (4,5).

Meanwhile, the increased clinical use of erythromycin and its derivatives, mostly in upper respiratory tract infections, has been related to an increased resistance of GAS to this antibiotic. Even though fewer than 5% of GAS isolates are reported as resistant to macrolide, lincosamide, and streptogramin (MLS) antibiotics (2,6), local exceptions have been reported, and widespread GAS resistance to erythromycin has so far been reported in Australia (17.6%) (7), Finland (20%) (8), the United Kingdom (22.8%) (9), Japan (60%) (10), and Taiwan (percentage not specified) (11).

Awareness of GAS resistance to erythromycin seems limited. Clinical microbiology laboratories rarely determine erythromycin susceptibility on a routine basis, and only recently have erythromycin breakpoints for streptococci other than *S. pneumoniae* been added in the latest National Committee for Clinical Laboratory Standards (NCCLS) document (12). Since the late 1980s, appreciable incidences of macrolide resistance in cases of pharyngotonsillitis and scarlet fever have also been reported from Italy (13-15).

Temporal trends in GAS resistance to erythromycin and clindamycin were systematically appraised on the basis of data collected over the last 3 years from 15 laboratories that participated in the Italian Surveillance Group for Antimicrobial Resistance (ISGAR). All the strains were isolated from throat swabs collected from symptomatic patients (mostly school-age outpatients) from 1993 through 1995. The number of isolates tested per year and the percentage of resistant ones are represented in the Figure.

GAS were identified by beta-hemolysis production on sheep or horse blood agar plates and by the presence of Lancefield group A antigen tested by commercial latex agglutination techniques (Streptex, Murex Diagnostics Ltd., Dartford, England; Phadebact, Boule Diagnostics AB, Huddinge, Sweden). The susceptibility tests used either the disk diffusion method (according to NCCLS performance standards [16,17]) or semiautomated microdilution tests (ATB, bioMérieux S.A., Marcy-l'Etoile, France; Sceptor, Becton Dickinson Diagnostic Instrument Systems, Sparks, Maryland), which were carried out as recommended by the respective manufacturers. The disk diffusion tests were read by manual measurement of the inhibition diameters or by a semi-automated system equipped with a video camera and image processing software that records the inhibition diameters (Bio-Videobact, Biokit S.A., Barcelona, Spain).

The data came from each automated reader device through data acquisition interfaces (created for that purpose by the respective manufacturers) and were subsequently translated through the *MyMic* software package (18) from individual proprietary formats into a common file format (Xbase) and transmitted to the reference center (Verona) on floppy disks or by electronic mail.

Test results were originally attributed to the different interpretive categories according to the NCCLS documents in force up untillate 1995 (16,17,19). After the data arrived in the reference center, they were reinterpreted on the basis of the new criteria for testing streptococcal species in the latest NCCLS document (12). The zone diameter criteria for resistant and susceptible isolates were 15 and 21, respectively, for erythromycin, and 15 and 19, respectively, for clindamycin. The equivalent minimum inhibitory concentration breakpoints (g/ml) for resistant and susceptible isolates were 4 and 0.5, respectively, for erythromycin, and 1 and 0.25, respectively, for clindamycin.

The survey showed a dramatic increase in the isolation of erythromycin-resistant strains of GAS (Figure). Both the rapid increase in the resistance rate in the areas involved and the subsequent involvement of other geographic sites caused considerable immediate concern since erythromycin had hitherto been effective against most isolates of this species and had been the drug of choice for treating streptococcal infections in patients allergic to penicillin.

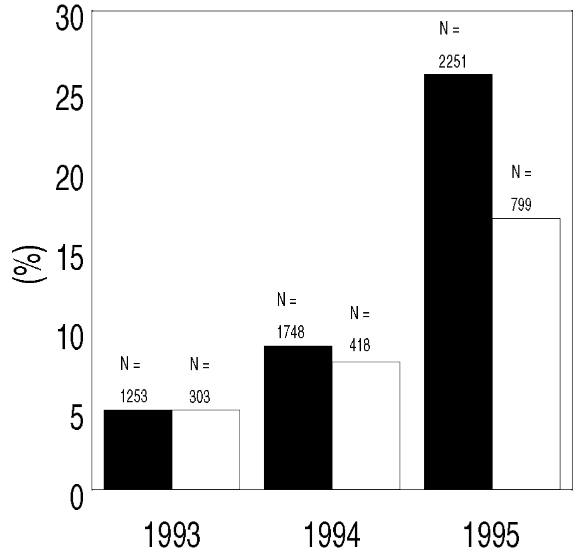

Figure. Number of GAS isolates tested per year and percentage of resistance to erythromycin and clindamycin, Italy, 1993-1995. Black bars represent erythromycin resistance and white bars, clindamycin resistance.

In 1993, the first year surveyed, the incidence of erythromycin-resistant strains was on average 5.1%, with marked variations according to geographic area, from 0% (all 19 strains from Pistoia) to 19.1% (Sassari, 18 strains out of 94). Two years later, in 1995, the incidence of resistant strains had registered a 1.5- to roughly 20-fold increase, with a mean value of 26.8%, from 13% (Palermo area, 3 strains out of 23) to 62% (Venice area, 31 strains out of 50). This incidence again showed geographic variations, but exceeded 40% in three centers out of 13 and 30% in another four. The Palermo area yielded the lowest rate of resistant strains, but its incidence of intermediate strains was exceptionally high (39.1%, 9 strains out of 23, versus a 5% to 10% rate in all other centers).

Resistance to clindamycin was more difficult to evaluate since this antibiotic was tested in only a few centers and on limited numbers of isolates. An increase in clindamycin resistance was recorded in five out of the seven centers that made this kind of data available. The highest rate of clindamycin resistance in 1995 was recorded in Verona (28.9%, 39 strains out of 135), while the lowest was recorded in Sassari (2.2%, one single strain out of 45). For all the strains studied, normal levels of susceptibility to penicillin and ampicillin were reported.

Molecular typing of erythromycin-resistant isolates was performed on strains isolated in the area of Verona in the first 2 months of 1995 (Table). Nine strains out of 14 were resistant to erythromycin, the 16-membered macrolide miokamycin, and the lincosamide clindamycin (the so-called $MLS_B$ phenotype, which has reduced binding of MLS antibiotics to their shared 50S rRNA target site [9,20]); the other five strains were resistant to erythromycin but not to miokamycin or clindamycin (the so-called M phenotype, in which resistance is attributed to an efflux system [21]). All strains of the

Table. Antibiotic susceptibilities,* presence of the *ermAM* gene, pulse-field gel electrophoresis type, and T-protein pattern of *Streptococcus pyogenes* strains isolated in Verona

| Strain | ERY† | MIO‡ | CLI§ | *ermAM* | PFGE type | T-protein pattern |
|---|---|---|---|---|---|---|
| VR1 | R | S | S | - | A | 8, 25 |
| VR2 | R | S | S | - | A | 8, 25 |
| VR3 | R | S | S | - | A | 25 |
| VR4 | R | S | S | - | A | 8, 25 |
| VR5 | R | R | R | + | B | 2, 28 |
| VR6 | R | R | R | + | C | 5, 12, 27 |
| VR7 | R | S | S | - | D | 4 |
| VR8 | R | R | R | + | C | 12, 27 |
| VR9 | R | R | R | + | E | 5, 12, 27 |
| VR10 | R | R | R | + | B | 2 |
| VR11 | R | R | R | + | C | 12, 27 |
| VR13 | R | R | R | + | C | 5, 12, 27 |
| VR14 | R | R | R | + | C | 12, 27 |
| VR15 | R | R | R | + | C | 5, 12, 27 |

*Resistant, susceptible
†Erythromycin
‡Miokamycin
§Clindamycin

$MLS_B$ phenotype carried the *ermAM* gene, which determines resistance to all $MLS_B$ antibiotics, as investigated by polymerase chain reaction (PCR) performed on total DNA (22), by using the following oligonucleotide primers (sequence 5' to 3') derived from the published sequence of the gene (23):

MLS1: AGAAACCGATACCGTTTACGA
MLS2: GGTCAATCGAGAATATCGTCA

The PCR studies used the control strain *Streptococcus sanguis* V736, which carries the *ermAM* gene in plasmid pVA736 (24). In contrast, all strains of the M phenotype were negative to the PCR analysis.

Five different DNA restriction profiles (Table) were found by pulse-field gel electrophoresis (PFGE) of genomic DNA fragments digested with SmaI (Boehringer, Mannheim, Germany), with C predominant. Three profiles were found among $MLS_B$ strains, and two among M strains; no profile was common to both $MLS_B$ and M strains.

Serologic analysis with T-protein-specific antisera (Institute of Sera and Vaccines, Prague, Czech Republic) showed seven T-types. Within each PFGE type, similar but not identical T-types were identified. Again, no T-type was common to both $MLS_B$ and M strains (Table).

Molecular typing results showed a great heterogeneity of erythromycin-resistant isolates in Verona: by combining the PFGE-type and the serotype, at least eight different clones could be identified.

The polyclonality of Verona isolates and the largely different rates of erythromycin- and clindamycin-resistance in most centers seem to confirm that the M phenotype of resistance has become fairly frequent (21,25). The diffusion of GAS strains resistant to erythromycin and susceptible to miokamycin and clindamycin implies that testing of erythromycin alone is no longer sufficient to assess the susceptibility of GAS to all MLS antibiotics, contrary to the claims made by Leclerq and Courvalin (26).

### Acknowledgments

We thank Becton Dickinson Italia S.p.A., biokit Italia s.r.l., and bioMérieux Italia S.p.A. for interfacing their instruments with the *MyMic* software; Andrea Di Clemente and Maurizio Trombini for their excellent technical assistance; and Anthony Steele for his help with the English language version of this paper. We dedicate this article to Giuseppe Satta, long-time promoter of the Italian Surveillance Group for Antimicrobial Resistance, who died 9 October 1994 at age 52.

**Giuseppe Cornaglia,* Marco Ligozzi,* Annarita Mazzariol,* Myriam Valentini,* Graziella Orefici,† the Italian Surveillance Group for Antimicrobial Resistance, ‡ and Roberta Fontana***

*Institute of Microbiology, University of Verona, Italy; †National Health Institute, Rome, Italy. ‡The Italian Surveillance Group for Antimicrobial Resistance: Antonio Goglio, Cristiana Passerini Tosi, Ospedali Riuniti di Bergamo; Massimo Gallina, Panaiota Troupioti, Azienda Ospedaliera 'Morelli', Bormio; Bruno Maranini, Riccardo Cioni, Ospedale 'San Giuseppe' Empoli; Pierluigi Nicoletti, Laura Martelli, Azienda Ospedaliera 'Careggi', Florence, Sergio Frugoni, Amelia Berlusconi, Pio Albergo Trivulzio, Milan; Roberto Rescaldani, Simone Bramati, Ospedale 'San Gerardo', Monza; Alfredo Chiarini, Anna Giammanco, Dipartimento di Igiene e Microbiologia, Università di Palermo; Roberto Rossetti, Spedali Riuniti, Pistoia; Gianfranco Santini, Annapaola Callegaro, Ospedale Civile, Pordenone; Giovanni Fadda, Teresa Spanu, Istituto di Microbiologia, Università Cattolica del Sacro Cuore, Rome; Alessandro Maida, Elena Muresu, Bianca Maria Are, Istituto di Igiene e Medicina Preventiva, Università di Sassari; Piero Cappuccinelli, Silvana

Sanna, Istituto di Microbiologia, Università di Sassari; Alessandro De Toffoli, Margherita Bergamasco, Ospedali Civili Riuniti, Venice; Mariuccia Scagnelli, Patrizia Reatto, Ospedale 'San Bortolo', Vicenza. Data manager: Giuseppe Cornaglia.

### References

1. Stevens DL. Streptococcal toxic shock syndrome: spectrum of disease, pathogenesis, and new concepts in treatment. Emerging Infectious Diseases 1995;1:69-78.
2. Kaplan EL. The resurgence of group A streptococcal infections and their sequelae. Eur J Clin Microbiol Infect Dis 1991;10:55-7.
3. Musher DM, Hamill RJ, Wright CE, Clarridge JE, Ashton CM. Trends in bacteremic infection due to *Streptococcus pyogenes* (group A streptococcus), 1986-1995. Emerging Infectious Diseases 1996;2:54-6.
4. Strömberg A, Romanus V, Burman LG. Outbreak of Group A streptococcal bacteremia in Sweden: an epidemiologic and clinical study. J Infect Dis 1991;164:595-8.
5. Martin PR, Höiby EA. Streptococcal serogroup A epidemic in Norway 1977-1988. Scand J Infect Dis 1990;22:421-9.
6. Duval J. Evolution and epidemiology of MLS resistance. J Antimicrob Chemother 1985;16:137-49.
7. Stingemore N, Francis GR, Toohey M, McGeechie DB. The emergence of erythromycin resistance in *Streptococcus pyogenes* in Fremantle, Western Australia. Med J Aust 1989;150:626-31.
8. Seppälä H, Nissinen A, Järvinen H, Huovinen S, Henriksson T, Herva E, et al. Resistance to erythromycin in Group A streptococci. N Engl J Med 1992;326:292-7.
9. Phillips G, Parratt D, Orange GV, Harper I, McEwan H, Young N. Erythromycin-resistant *Streptococcus pyogenes*. J Antimicrob Chemother 1990;25:723-4.
10. Maruyama SH, Yoshioka H, Fujiita K, Takimoto M, Satake Y. Sensitivity of group A streptococci to antibiotics: prevalence of resistance to erythromycin in Japan. Am J Dis Child 1979;133:1143-5.
11. Hsueh P-R, Chen H-M, Huang A-Y, Wu J-J. Decreased activity of erythromycin against *Streptococcus pyogenes* in Taiwan. Antimicrob Agents Chemother 1995;39:2239-42.
12. National Committee for Clinical Laboratory Standards. Performance standards for antimicrobial susceptibility testing: sixth informational supplement. National Committee for Clinical Laboratory Standards document M100-S6. National Committee for Clinical Laboratory Standards, Villanova, Pa. 1995.
13. Borzani M, Varotto F, Garlaschi L, Conio F, Dell'Olio M, Careddu P. Clinical and microbiological evaluation of miocamycin activity against group A in pediatric patients: three years' incidence of erythromycin resistant group A streptococci. J Chemother 1989;1:35-8.
14. Scopetti F, Pataracchia M, Pacifico L, Ranucci A, Morini C, Cherchi G, et al. A multicenter study on antimicrobial susceptibility and typing of group A streptococci from cases of pharyngo-tonsillitis and scarlet fever. Microecology and Therapy 1995;25:348-55.
15. Cellesi C, Chigiotti S, Zanchi A, Mencarelli M, Corbisiero R, Rossolini GM. Susceptibility to macrolide and beta-lactam antibiotics of *Streptococcus pyogenes* strains isolated over a four-year period in central Italy. J Chemother 1996;8:188-92.
16. National Committee for Clinical Laboratory Standards. Methods for dilution antimicrobial susceptibility tests for bacteria that grow aerobically, M7-A3. National Committee for Clinical Laboratory Standards, Villanova, Pa. 1993.
17. National Committee for Clinical Laboratory Standards. Performance standards for antimicrobial disk susceptibility tests, M2-A5. National Committee for Clinical Laboratory Standards, Villanova, Pa. 1993.
18. Cornaglia G, Satta G. *MyMic*: computerised listing of antimicrobials with optimal activity and pharmacokinetic properties for individual infections. Binary 1993;5:159-64.
19. National Committee for Clinical Laboratory Standards. Performance standards for antimicrobial susceptibility testing: fifth informational supplement. National Committee for Clinical Laboratory Standards document M100-S5. National Committee for Clinical Laboratory Standards, Villanova, Pa. 1994.
20. Weisblum B. Erythromycin resistance by ribosome modification. Antimicrob Agents Chemother 1995;39:577-85.
21. Sutcliffe J, Tait-Kamradt A, Wondrack L. *Streptococcus pneumoniae* and *Streptococcus pyogenes* resistant to macrolides but sensitive to clindamycin: a common resistance pattern mediated by an efflux system. Antimicrob Agents Chemother 1996;40:1817-24.
22. Arthur M, Molinas C, Mabilat C, Courvalin P. Detection of erythromycin resistance by the polymerase chain reaction using primers in conserved regions of *erm* rRNA methylase genes. Antimicrob Agents Chemother 1990;34:2024-6.
23. Arthur M, Brisson-Noël A, Courvalin P. Origin and evolution of genes specifying resistance to macrolide, lincosamide and streptogramin antibiotics: data and hypothesis. J Antimicrob Chemother 1987;20:783-802.
24. Macrina FL, Jones KR, Wood PH. Chimeric plasmids and their use as molecular cloning vehicles in *Streptococcus sanguis* (Challis). J Bacteriol 1980;143:1425-35.
25. Seppälä H, Nissinen A, Yu Q, Huovinen P. Three different phenotypes of erythromycin-resistant *Streptococcus pyogenes* in Finland. J Antimicrob Chemother 1993;32:885-91.
26. Leclerq R, Courvalin P. Bacterial resistance to macrolide, lincosamide and streptogramin antibiotics by target modification. Antimicrob Agents Chemother 1991;35:1267-72.
